# Long-term Subclinical Cardiotoxicity of Modern Cardiotoxic Treatment Protocols in Childhood Cancer Survivors Assessed by Cardiovascular Magnetic Resonance T1 Mapping and Circulatory Biomarkers

**DOI:** 10.1007/s12012-026-10098-8

**Published:** 2026-01-28

**Authors:** Roman Panovský, Marie Tomandlová, Mary Luz Mojica-Pisciotti, Tomáš Kepák, Jan Máchal, Věra Feitová, Jan Frič, Marcela Hortová-Kohoutková, Tomáš Holeček, Josef Tomandl, Lukáš Opatřil, Vladimír Kincl

**Affiliations:** 1 International Clinical Research Center, St. Anne’s University Hospital Brno, Pekařská 53, 60200 Brno, Czech Republic; 2 1 st Department of Internal Medicine/Cardioangiology, St. Anne’s University Hospital Brno, Pekařská 53, 60200 Brno, Czech Republic; 3 Faculty of Medicine, Masaryk University, Kamenice 5, 62500 Brno, Czech Republic; 4 Department of Biochemistry, Faculty of Medicine, Masaryk University, Kamenice 5, 62500 Brno, Czech Republic; 5 Department of Children’s Oncology, University Hospital Brno, Masaryk University, Černopolní 9, 61300 Brno, Czech Republic; 6 Department of Pathophysiology, Faculty of Medicine, Masaryk University, Kamenice 5, 62500 Brno, Czech Republic; 7 Department of Medical Imaging, St. Anne’s University Hospital Brno, Pekařská 53, 60200 Brno, Czech Republic; 8 Institute of Hematology and Blood Transfusion, U Nemocnice 1, 12800 Prague, Czech Republic; 9 Department of Biomedical Engineering, Brno University of Technology, Technicka 12, 61600 Brno, Czech Republic

**Keywords:** Cardiac magnetic resonance, Childhood cancer survivors, Anthracyclines, T1 mapping, Circulatory biomarkers

## Abstract

Childhood cancer survivors (CCS) are at increased risk of developing heart disease due to the cardiotoxic effects of oncological treatment. This study aimed to investigate the long-term cardiotoxic effects of cancer therapy in CCS using a multimodal approach combining cardiac magnetic resonance (CMR) imaging and circulating blood biomarkers. A total of 117 CCS (mean age 24.7 ± 5.2 years), at least five years post-treatment and in complete remission, were prospectively enrolled. All participants underwent CMR, including T1 mapping, and blood analysis for biomarkers of endothelial damage and oxidative stress. Parameters were compared with sex- and age-matched healthy control groups. Anthracycline treatment was administered in 82.9% of CCS (mean cumulative doxorubicin equivalent dose 231.7 ± 92.0 mg/m²). Left ventricular ejection fraction and mitral annular plane systolic excursion were significantly reduced in CCS compared to controls (59.0 ± 5.5% vs. 67.2 ± 6.9%, *p* < 0.001; 12.5 ± 1.7 mm vs. 13.9 ± 2.2 mm, *p* = 0.001). Late gadolinium enhancement was detected in four CCS. No significant differences in global native T1 relaxation time or extracellular volume were observed. N-terminal pro-B-type natriuretic peptide (NT-proBNP) levels remained within normal limits but correlated with native T1, ECV, and MAPSE. Levels of myeloperoxidase, big endothelin-1, and ischemia-modified albumin were significantly higher than in controls. Subclinical myocardial changes were detected in long-term CCS using CMR and circulating biomarkers. These findings support the utility of a multimodal approach for the early identification of individuals at increased cardiovascular risk after childhood cancer treatment.

**Supplementary Information:**

The online version contains supplementary material available at 10.1007/s12012-026-10098-8.

## Background

Due to advancements in oncological therapies, the long-term survival rate of childhood cancer survivors (CCS) now exceeds 80%, leading to a steadily growing population of CCS. However, up to 60% of CCS were exposed to cardiotoxic treatments, including anthracyclines and radiation exposure to the heart, and have shown to have a significantly increased risk of developing congestive heart failure (CHF) [1, 2]. CHF has become one of the leading causes of long-term morbidity and mortality in this vulnerable population [1, 3–6], with CCS facing a 15-fold higher risk of developing CHF [4] and a 7-fold increased risk of premature cardiac-related death compared with the general population [7].

Anthracyclines, such as doxorubicin, daunorubicin, idarubicin, and epirubicin, are widely used in pediatric oncology. All anthracyclines have toxic effects on the myocardium. Compared to anthracycline-free protocols, the incidence of both subclinical and clinical cardiotoxicity is markedly higher, with reported rates of 2–5% in breast cancer survivors [8–11] and even higher among CCS [3, 4, 12, 13]. The risk of cardiotoxicity is dose-dependent and modulated by additional factors, including age at treatment, sex, and concomitant therapies. Although the introduction of dose-reduction strategies has led to a decline in cancer therapeutics-related cardiac dysfunction (CTRCD) to about 1–3%, even low cumulative anthracycline exposure can cause irreversible myocardial alterations [14, 15].

Cardiac magnetic resonance (CMR) has the potential to detect early phases of cardiac involvement, allowing for the possibility of taking preventive steps against the development of advanced stages of CHF. CMR includes highly accurate and reproducible techniques and is the gold standard for evaluating volume, myocardial mass (MM), and function of the left (LV) and right ventricles (RV). CMR is an integral part of the diagnostic algorithm for CCS, as recommended by the ESC guidelines [16]. Compared to other imaging methods, it can depict the structure of the myocardium and show even a small myocardial muscle damage. Among the CMR techniques, late gadolinium enhancement (LGE) offers a unique possibility to assess myocardial structure in vivo, especially for identifying fibrosis. Additionally, T1 mapping facilitates the detection of very early cardiac involvement, even before any decline in the left ventricular ejection fraction (LVEF) becomes evident. However, contradictory results from studies using T1 mapping in CCS monitoring have been published [17–28].

Moreover, circulating blood biomarkers can offer complementary insight into sub-clinical cardiotoxicity. Particularly promising are markers reflecting endothelial dysfunction or oxidative stress—two pathophysiological mechanisms strongly implicated in chemotherapy-induced cardiac injury. Disruption of physiological endothelial health is believed to be a key contributor to cardiotoxicity. Once administered, chemotherapeutic agents interact with vascular endothelial cells on the luminal surface of blood vessels, making them highly vulnerable and resulting in disruption of endothelial physiology and dysfunction [29]. The situation is more complicated with oxidative stress, as it cannot be only the cause of endothelial dysfunction, but also a consequence of endothelial dysfunction that may have been induced by treatment [30]. Alternatively, chemotherapeutic agents and endothelial dysfunction are closely connected to inflammation and inflammatory cytokines, further contributing to cardiotoxicity [31]. We have shown earlier that the immune system of CCS normalises only after several years of therapy [32] and that inflammation is a critical concern in CCS cohorts [33].

This study aims to evaluate long-term cardiotoxicity in CCS by integrating CMR techniques, including T1 mapping, with circulating blood biomarkers.

## Methods

### Patient Population

The study cohort included 117 CCS between 18 and 40 years old who were at least 5 years off treatment and in complete remission were included. Criteria for inclusion were: 1/signed informed consent; 2/no MR contraindications (e.g. implanted pacemaker/defibrillator, cochlear implant or another ferromagnetic material in the body, claustrophobia); 3/no contraindications for gadolinium contrast agent administration (renal failure); 4/ability of the patient to cooperate during the MR examination; 5/absence of any known cardiac disease; 6/absence of another oncological disease in patient’s history; and 7/absence of any autoimmune disease in patient’s history. All included CCS underwent CMR examination and laboratory tests. A sex- and age-matched control group for comparison of CMR measures (imaging control group, CG1) was built from our database of subjects with a clinical indication for CMR (to exclude cardiac disease due to nonspecific syndromes) with no relevant cardiac medical history or symptoms, normal cardiac investigation results, and normal CMR findings. Similarly, sex- and age-matched healthy volunteers served as a control group for the biochemistry part (biomarkers control group, CG2). Healthy blood donors were collected from the Department of Transfusion and Tissue Medicine, University Hospital, Brno (Czech Republic). Analyses were conducted both for the entire cohort and separately for the subgroup of CCS treated with anthracyclines (CCS-A). The cumulative doxorubicin equivalent dose was calculated according to standard conversion formulas, taking into account the relative cardiotoxicity of different anthracyclines [34, 35].

This prospective study was performed in accordance with the Declaration of Helsinki (2000) of the World Medical Association and approved by the institutional ethics committee (Ethics Committee of St Anne’s University Hospital Brno, reference number 15 V/2018). Written informed consent was obtained from all the subjects.

### CMR Data Acquisition

All CCS and controls underwent a complete CMR examination at a 1.5T scanner (Ingenia, Philips Medical Systems) following our standard protocol [36]. The CMR examination included cine, native and postcontrast T1 parametric mapping, and delayed postcontrast images. Cine images were obtained with balanced turbo field echo steady-state free precession (SSFP) sequences (typical parameters: field of view 300 × 300 mm, acquisition voxel size 1.67 × 1.67 × 8.00 mm, reconstruction matrix 256, slice thickness 8 mm, SENSE factor 1.7, 30 to 50 frames per cardiac cycle) in long-axis (two-chamber, four-chamber, three-chamber) and short-axis (SAX) views.

T1 mapping was performed using a modified Look-Locker inversion recovery sequence (MOLLI) in the mid-ventricular level in the short-axis plane before (native) and 15 min after contrast agent administration (enhanced). A 3(3)5 MOLLI scheme for native T1 mapping and 4(1)3(1)2 for enhanced T1 mapping were used.

LGE images in all long-axis views and the SAX stack were acquired 10 min after an intravenous bolus of a total of 0.2 mmol/kg of the gadolinium-based contrast agent gadobutrol (Gadovist, Bayer-Schering Pharma, Germany) using an inversion-recovery turbo field echo sequence (IR-TFE). In case of doubt, phase-sensitive inversion recovery (PSIR) TFE images were acquired. Both 2-dimensional and 3-dimensional data acquisitions were performed.

### CMR Data Analysis

Regional wall motion abnormalities were assessed visually using cine images. The LV volumetric and functional parameters were calculated from the cine SAX stack using the summation-of-disc methods following the recommendations on post-processing evaluation from the Society for Cardiovascular Magnetic Resonance [37] on the IntelliSpace Portal (ISP) workspace (version 11, Philips Healthcare, Best, The Netherlands). The reported variables included the left ventricle ejection fraction (LVEF), LV end-diastole volume (LVEDV), LV end-systole volume (LVESV), LV stroke volume (LVSV), LV mass (LVMM), and mitral annular plane systolic excursion (MAPSE) as a mean of MAPSE from the septal and lateral parts of the mitral annulus. Volumes and MM were indexed by BSA (body surface area). LGE was assessed visually as an area of visually identified contrast enhancement higher than the mean signal intensity of the reference myocardium.

The T1 parametric mapping analysis, including assessment of the extracellular volume (ECV), was performed using dedicated software cvi42 (v5.13.9, Circle Cardiovascular Imaging, Calgary, Canada). The T1 native, T1 enhanced, and ECV values were calculated for each of the six AHA-defined segments of the mid-ventricular SAX slice. Global values were the average in those segments. The hematocrit was obtained closely before the CMR examination.

### Laboratory Tests

Markers reflecting endothelial damage—Big endothelin-1 (bigET-1) and thrombomodulin (TM), and oxidative stress—ischemia-modified albumin (IMA) and myeloperoxidase (MPO), were evaluated. Venous blood samples were collected on the same day as the CMR examination in the morning. Immediately after centrifugation, serum and EDTA-plasma samples were aliquoted and subsequently stored at − 80 °C until analysis. Biochemical blood tests were performed within one to four months, except for N-terminal B-type natriuretic peptide (NT-proBNP), which was determined only for CCS on the day of collection as part of routine laboratory testing and subsequently evaluated against established reference ranges, i.e., 0–300 pg/ml.

Serum levels of IMA were determined using the colorimetric method Co(II)-albumin binding assay as described by Bar-Or [38]. The limit of quantification was 0.010 absorbance unit (ABSU), and the intra- and inter-assay coefficient of variation (CV) (albumin 32.3 g/L) were 1.7% and 6.8%, respectively. Levels of bigET-1 were measured using a sandwich enzyme-linked immunosorbent assay (ELISA) (Biomedica, Austria); the limit of detection was 0.02 pmol/L, with an intra-assay CV ˂ 5% and an inter-assay CV ˂ 4%. Plasma levels of MPO were measured using a commercially available ELISA kit (Immundiagnostik AG, Germany), and the limit of detection was 2.9 ng/mL, with the intra- and inter-assay CV < 3% and < 6%, respectively. Serum TM levels were determined using an ELISA kit (R&D Systems, MN, USA) according to the manufacturer’s protocol; the limit of detection is 78.2 pg/mL, and the intra- and inter-assay CVs were < 4% and < 8%, respectively.

### Statistical Analysis

Welch’s t-test was used to compare the normally distributed data that were previously tested by the Kolmogorov-Smirnov test and visual inspection of histograms (i.e., generally the results of imaging methods), the Mann-Whitney U-test was used for other distributions and discrete data (i.e., laboratory results and anthracycline dose), and the two-tailed Fisher exact test was used for binary data. Spearman’s rank coefficient was employed to assess the correlations among the quantitative variables. The value of α = 0.05 was used throughout the analysis. The calculations were performed using Statistica software (TIBCO Software, v14.0.0.1.).

## Results

**Fig. 1:**
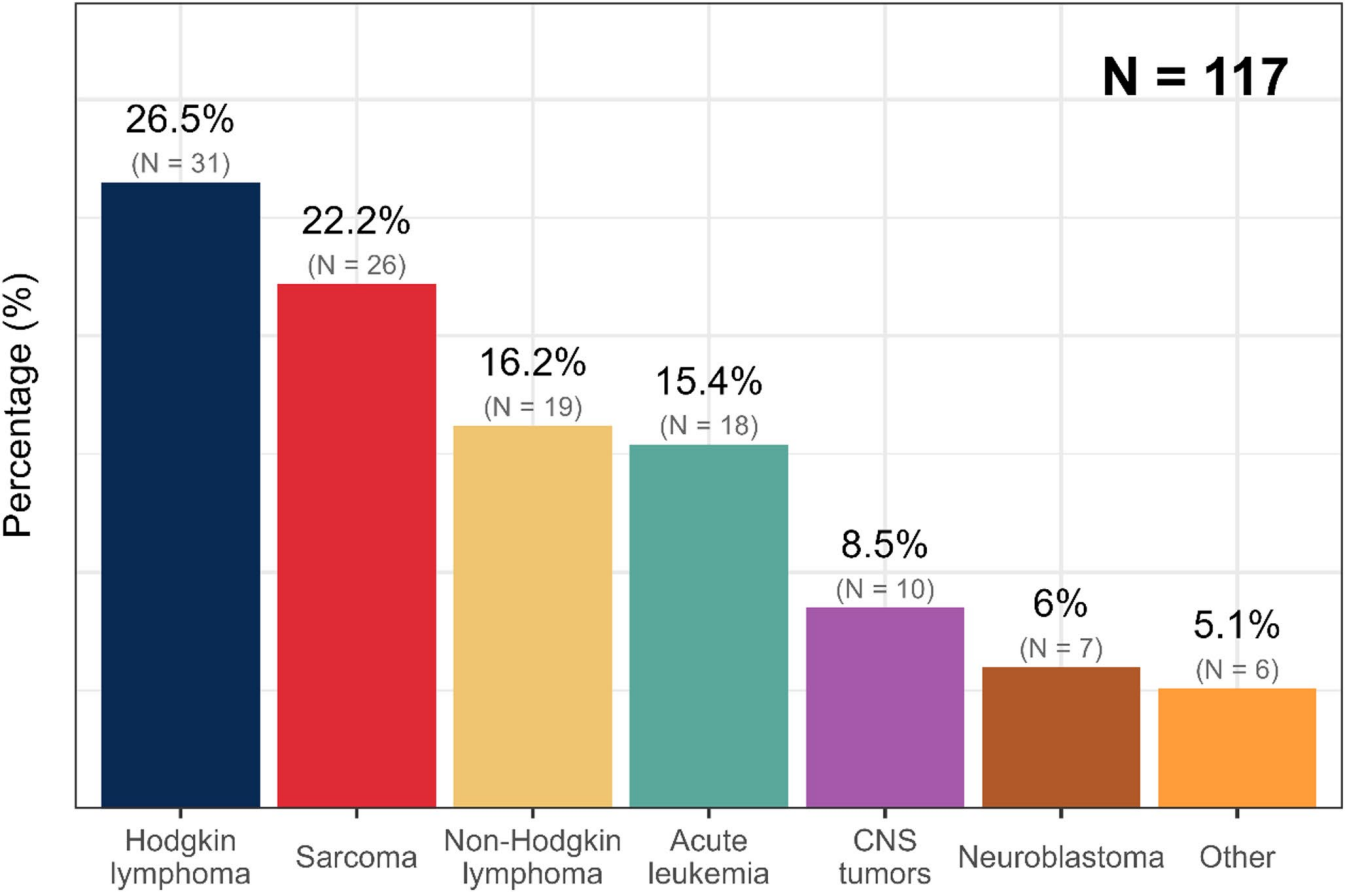
Distribution of cancer types in the child cancer survivors cohort; *CNS* central nervous system

Together, 117 CCS (mean age 24.7 ± 5.2 years), 39 controls of the CG1 group (imaging control group) (26.9 ± 7.9 years, *p* = 0.045), and 20 controls of the CG2 group (biomarkers control group) (24.6 ± 3.4 years, *p* = 0.656) were included (Table 1). The most frequent diagnoses of CCS were Hodgkin lymphoma (*n* = 31, 26.5%), sarcoma (*n* = 26, 22.2%), non-Hodgkin lymphoma (*n* = 19, 16.2%), and acute leukemia (*n* = 18, 15.4%) (Fig. 1). The mean time since the end of cancer-therapeutics treatment was 12.2 ± 5.8 years. All CCS underwent chemotherapy: 90 (76.9%) were treated with doxorubicin, 21 (17.9%) with daunorubicin, 6 (5.1%) with epirubicin, 6 (5.1%) with cisplatin, and 3 (2.6%) with carboplatin. Together, 97 (82.9%) CCS were treated with anthracyclines (Table 1). The average dose of isotoxic equivalent of doxorubicin was 231.7 ± 92.0 mg/m^2^. Most CCS were treated with a low dose of anthracycline; only 27 (23.0%) received a dose equivalent of doxorubicin higher than 300 mg/m^2^. Sixty-six CCS had radiation therapy, 47 of them to the thoracic region, and 27 subjects underwent stem cell therapy.

**Table 1:** Clinical parameters for the study cohort for CCS, including those treated by anthracyclines (CCS-A), the imaging control group (CG1), and the biomarkers control group (CG2)

Parameter	CCS (*n* = 117)	Anthracycline-treated CCS (CCS-A) (*n* = 97)	CG1 (*n* = 39)	CG2 (*n* = 20)	*p*-value CCS v CG1	*p*-value CCS v CG2	*p*-value CCS-A v CG1	*p*-value CCS-A v CG2
Age [years]	24.7 ± 5.2	24.2 ± 4.1	26.9 ± 7.9	24.6 ± 3.5	0.104	0.920	**0.043**	0.615
Female [n (%)]	45 (38.5%)	37 (38.1%)	13 (33.3%)	8 (40.0%)	0.702	1.000	0.695	1.000
BMI [kg/m^2^]	22.7 ± 4.0	22.7 ± 3.9	23.7 ± 3.6	24.3 ± 3.2	0.185	0.053	0.145	**0.047**

Bold values indicate statistically significant differences (*p* < 0.05)

Variables are expressed as numbers/total (percentages) or mean (standard deviation) for categorical, normally distributed, and non-normally distributed continuous variables. *CCS* childhood cancer survivors, *CG1* control group 1 (imaging control group), *CG2* control group 2 (biomarkers control group), *n* total number of subjects, *BMI* body mass index

LV regional and global systolic function was almost within normal limits in all CCS; only 2 (1.7%) subjects had an LVEF under 50% (49% and 48%), and 3 (2.6%) had moderate hypokinesis of the LV wall (1 in the inferior wall, 1 in the interventricular septum, and 1 global). Nevertheless, the mean LVEF was lower in CCS compared to CG1 (59.0 ± 5.5% vs. 67.2 ± 6.9%, *p* < 0.001). Also, mean MAPSE was slightly decreased in CCS (12.5 ± 1.7% vs. 13.9 ± 2.2%, *p* = 0.001). Significantly higher EDVi (74.8 ± 14.0 ml/m^2^ vs. 65.0 ± 9.3 ml/m^2^, *p* < 0.001) were found in the CCS-A group, while lower LVMMi was measured in CCS (39.5 ± 11.5 g/m^2^ vs. 50.4 ± 14.5 g/m^2^, *p* < 0.001) (Table 2). Only 4 (6%) CCS had LGE in the myocardium, all of which was small and patchy LGE in the interventricular septum. One CCS (0.9%) had LGE of thickened pericardium. The subgroup treated with chest radiotherapy (*n* = 47) differed in left ventricle morphology and function compared to those who did not receive chest radiotherapy (*n* = 70), as shown in Supplementary Table 1.

**Table 2:** Left ventricular functional and volumetric parameters for CCS, including those treated by anthracyclines (CCS-A), and the imaging control group (CG1)

Parameter	CCS (*n* = 117)	Anthracycline-treated CCS (CCS-A) (*n* = 97)	CG1 (*n* = 39)	*p*-value CCS v CG1	*p*-value CCS-A v CG1
LVEF (%)	59.0 (5.5)	58.5 (5.2)	67.2 (6.9)	**< 0.001**	**< 0.001**
LVEDV (ml)	131.9 (30.6)	134.6 (30.1)	123.5 (23.9)	0.082	**0.026**
LVESV (ml)	54.4 (15.8)	56.2 (15.9)	40.9 (12.2)	**< 0.001**	**< 0.001**
LVSV (ml)	77.6 (18.3)	78.5 (17.5)	82.6 (16.7)	0.124	0.215
LVMM (g)	72.3 (25.1)	72.2 (22.9)	96.9 (34.6)	**< 0.001**	**< 0.001**
LVEDVi (ml/m^2^)	73.6 (14.3)	74.8 (14.0)	65.0 (9.3)	**< 0.001**	**< 0.001**
LVESVi (ml/m^2^)	30.3 (7.7)	31.2 (7.8)	21.4 (5.2)	**< 0.001**	**< 0.001**
LVSVi (ml/m^2^)	43.4 (8.7)	43.7 (8.3)	43.6 (7.4)	0.857	0.970
LVMMi (g/m^2^)	39.5 (11.5)	39.3 (10.9)	50.4 (14.5)	**< 0.001**	**< 0.001**
MAPSE (mm)	12.5 (1.7)	12.6 (1.6)	13.9 (2.2)	**0.001**	**0.002**

Bold values indicate statistically significant differences (*p* < 0.05)

Variables are expressed as mean (standard deviation), *p*-values correspond to Welch’s *t*-test. Abbreviations: *CCS* childhood cancer survivors, *CCS-A* childhood cancer survivors treated by anthracyclines, *CG1* control group 1 (imaging control group), *n* total number of subjects, *EF* ejection fraction; *EDV* end-diastole volume, *ESV* end-systole volume, *i* indexed, *LV* left ventricle, *MM* myocardial mass, *MAPSE* mitral annular plane systolic excursion, *SV* stroke volume

Local and global native T1 times and ECV are shown in Table 3. There were no significant differences in native T1 and ECV between CCS or CCS-A and CG1, with the exception of significantly higher native T1 value in the anteroseptal segment in CCS compared to CG1 (996.9 ± 36.1 ms vs. 986.3 ± 25.4 ms, *p* = 0.046).

**Table 3:** T1 mapping analysis: T1 native and ECV for CCS, including those treated by anthracyclines (CCS-A), and the imaging control group (CG1)

Parameter	CCS (*n* = 117)	Anthracycline-treated CCS (CCS-A) (*n* = 97)	CG1 (*n* = 39)	*p*-value CCS v CG1	*p*-value CCS-A v CG1
T1 native (ms)					
Anterior	994.4 (36.3)	993.1 (37.0)	984.5 (30.1)	0.095	0.165
Anteroseptal	996.9 (36.1)	997.3 (38.3)	986.3 (25.4)	**0.046**	0.052
Inferoseptal	998.8 (33.2)	998.5 (34.8)	990.9 (28.2)	0.151	0.186
Inferior	992.4 (35.5)	991.7 (36.7)	983.6 (30.6)	0.140	0.190
Inferolateral	993.8 (41.3)	992.0 (40.8)	990.5 (35.1)	0.623	0.822
Anterolateral	985.4 (33.1)	984.5 (32.8)	976.5 (33.7)	0.157	0.211
Global	994.4 (30.2)	993.6 (31.4)	986.0 (25.6)	0.095	0.147
**ECV (%)**					
Anterior	25.3 (3.0)	25.5 (3.0)	25.1 (3.1)	0.605	0.466
Anteroseptal	25.5 (3.0)	25.7 (2.9)	25.5 (3.1)	0.990	0.787
Inferoseptal	25.7 (2.9)	25.9 (2.8)	25.5 (2.9)	0.671	0.453
Inferior	25.1 (3.0)	25.2 (3.0)	24.8 (3.1)	0.553	0.441
Inferolateral	24.2 (2.9)	24.2 (2.8)	24.2 (2.9)	0.866	0.878
Anterolateral	24.6 (2.9)	24.7 (2.8)	24.6 (3.1)	0.871	0.780
Global	25.1 (2.7)	25.2 (2.7)	25.0 (2.8)	0.743	0.599

Bold values indicate statistically significant differences (*p* < 0.05)

Variables are expressed as mean (standard deviation), *p*-values correspond to Welch’s *t*-test. Abbreviations: CCS, childhood cancer survivors; *CCS-A* childhood cancer survivors treated by anthracyclines, *CG1* control group 1 (imaging control group), *n* total number of subjects, *T1* T1 relaxation time, *ECV* extracellular volume fraction

There was a significant correlation between LVEF and cumulative doxorubicin equivalent (*r* = − 0.21, *p* = 0.043), and also a non-significant correlation for LVEDV (*r* = − 0.17, *p* = 0.088). On the contrary, no correlation was found between the cumulative doxorubicin equivalent and the T1 global native time (*r* = − 0.59, *p* = 0.550) and ECV (*r* = − 2.07, *p* = 0.329).

In routine laboratory testing, NT-proBNP levels in CCS were within normal limits (38 (22–81) pg/ml), as well as for those treated with anthracyclines (40 (20–77) pg/ml); none of the survivors had levels exceeding 300 pg/ml. Despite this fact, there were significant correlations between NT-proBNP level and global native T1, global ECV, and MAPSE (*r* = 0.31, *p* < 0.001; *r* = 0.50, *p* < 0.001; *r* = − 0.21, *p* = 0.021). Studied biomarker levels are summarized in Table 4. The concentrations of MPO, bigET-1, and IMA levels were significantly higher compared to CG2 (*p* < 0.001; 0,018; <0.001, respectively). Similar results were obtained by comparing MPO, bigET-1, and IMA in CCS-A with GC2 (*p* < 0.001; 0.035; <0.001, respectively).

**Table 4:** Comparison of biomarkers levels in childhood cancer survivors and controls for CCS, including those treated by anthracyclines (CCS-A), and the biomarkers control group (CG2)

Biomarker	CCS(n = 117)	Anthracycline-treated CCS (CCS-A)(n = 97)	CG2(n = 20)	p-value CCS v CG2	p-value CCS-A v CG2
Thrombomodulin (pg/mL)	4097 (3763; 4570)	4048 (3742; 44960)	4581 (3941; 4868)	0.127	0.074
Myeloperoxidase (ng/mL)	61.8 (53.9; 84.9)	62.0 (53.9; 84.9)	29.1 (28.0; 32.2)	**<0.001**	**<0.001**
Big endothelin-1 (pmol/L)	0.300 (0.210; 0.434)	0.293 (0.200; 0.410)	0.224 (0.164; 0.315)	**0.018**	**0.035**
IMA (ABSU)	0.372 (0.329; 0.406)	0.371 (0.331; 0.406)	0.258 (0.205; 0.297)	**<0.001**	**<0.001**

Bold values indicate statistically significant differences (*p* < 0.05)

Variables are expressed as median (interquartile range); *p*-values of the Mann–Whitney *U*-test are reported. Abbreviations: *ABSU* absorbance unit, *CCS* childhood cancer survivors, *CCS-A* childhood cancer survivors treated by anthracyclines, *CG2* control group 2 (biomarkers control group), *n *total number of subjects, *IMA* ischemia-modified albumin

The concentrations of thrombomodulin, MPO, bigET-1, and IMA did not differ between CCS who were or were not treated by chest radiotherapy (*p* > 0.10 in all cases). In CCS treated with anthracyclines (CCS-A), thrombomodulin showed a positive correlation with LVESV (*r* = 0.18, *p* = 0.046) and a negative correlation with LVEF (*r* = − 0.19, *p* = 0.041) and ECV (*r* = − 0.36, *p* < 0.001). Contrary to that, IMA showed a positive correlation with ECV (*r* = 0.35, *p* < 0.001) and global native T1 values (*r* = 0.20, *p* = 0.036), and a negative correlation with LVSVi (*r* = − 0.24, *p* = 0.011), LVMM (*r* = − 0.20, *p* = 0.033) and LVMMi (*r* = − 0.24, *p* = 0.011). MPO correlated positively with LVEDV (*r* = 0.22, *p* = 0.017) and LVSV (*r* = 0.23, *p* = 0.015) and negatively with global native T1 (*r* = − 0.24, *p* = 0.010) and ECV (*r* = − 0.18, *p* = 0.049). BigET-1 did not correlate with any of the parameters.

## Discussion

To the best of our knowledge, this is the first study to assess late cardiotoxicity in a population of CCS using a combination of CMR T1 mapping and circulating blood biomarkers reflecting endothelial damage and oxidative stress. The study highlights three crucial findings. First, CCS had elevated levels of blood markers, especially MPO, bigET-1, and IMA. Second, contrary to the laboratory test results, CMR imaging revealed just rare and minimal signs of possible cardiac damage. And third, there were no significant differences in the global native T1 and ECV between CCS and CG1.

Evaluation of blood markers may complement imaging techniques and potentially facilitate the detection of subclinical cardiotoxicity. In our study, bigET-1 and TM (reflecting endothelial damage) and IMA and MPO (reflecting oxidative stress) were measured. Although, given the time that has elapsed since treatment (at least 5 years), the levels of the monitored biomarkers may also be influenced by other circumstances, such as lifestyle or epigenetic factors, our results suggest that therapy could be a risk factor associated with increased levels of bigET-1, IMA, and MPO. Statistically significantly higher levels of these markers were found in CCS compared to CG2.

High plasma concentrations of bigET-1, a precursor protein of ET-1, have been associated with endothelial dysfunction and increased cardiovascular risk [39]. To our knowledge, an increase in bigET-1 has not been observed in CCS to date, but Romano et al. [40] recently reported a rise in ET-1 levels in brain cancer survivors. In animal models, doxorubicin has also been shown to increase plasma ET-1 concentrations [41].

MPO, an enzyme produced by polymorphonuclear leukocytes, has atherogenic and pro-oxidant effects on cardiac tissue, leading to its association with increased risk of coronary artery disease and acute heart failure [42, 43]. Dean et al. monitored the MPO levels before, after the last dose of, and 3–6 months after completion of doxorubicin chemotherapy and found that MPO levels 3–6 months after chemotherapy were significantly lower than before chemotherapy. This may be due to the high baseline MPO level caused by systemic inflammation related to cancer in some patients [44]. Our results suggest that, despite the aforementioned decrease in MPO levels over time, its levels can remain elevated in the CCS population.

IMA is human serum albumin that has been modified at its N–terminal end by free radicals [45]; serum IMA concentration is sometimes used as a marker of ischemia injury [46]. IMA has been studied as a marker of acute cardiotoxicity [47, 48]. Ma et al. demonstrated that its increase correlates with the cumulative doxorubicin dose and is useful for predicting long-term damage to cardiac function [48]. Our findings also showed an increased IMA in CCS compared to controls. On the other hand, our study did not reveal any differences in the levels of TM, a marker reflecting endothelial cell damage, between CCS and controls. This observation is consistent with the results of a previously published study by Sadurska et al., which also evaluated late cardiotoxicity [49].

Our study demonstrates that the LV systolic function in CCS is generally well preserved, with only 2 subjects (1.7%) exhibiting an LVEF below 50% (49% and 48%). However, the mean LVEF was significantly lower in CCS compared to controls, suggesting the presence of subclinical LV dysfunction despite overall preserved function. This finding aligns with previous research indicating that most CCS maintain global systolic function in the long term after treatment. Brouwer et al. [50] assessed systolic and diastolic function in 277 adult CCS and 130 healthy controls. They found that while CCS had preserved systolic function, they exhibited an increased prevalence of diastolic dysfunction compared to controls. Similarly, Wolf et al. [51] evaluated 79 CCS and found that despite normal LVEF, survivors exhibited subclinical cardiac dysfunction, correlating with cumulative anthracycline dose. In a systematic review, Merkx et al. [52], based on 11 studies, concluded that LVEF < 50% was observed in 1–6% of CCS. Reported risk factors were cumulative anthracycline dose and radiotherapy involving the heart region, with no safe dose for asymptomatic LV systolic dysfunction.

Similarly, the slightly reduced MAPSE observed in CCS may reflect early regional systolic abnormalities not fully captured by LVEF measurement alone. The absence of significant LV dilatation combined with a lower LVMM suggests myocardial remodelling characterized by atrophy or reduced muscle mass, potentially as a consequence of oncological treatment or its metabolic effects, as demonstrated in previous studies on CCS [20, 53].

LGE was detected in a small proportion of CCS (*n* = 4, 6.0%), with findings limited to patchy areas in the interventricular septum, consistent with minimal focal fibrosis or myocardial injury. Most studies in CCS report a low prevalence of LGE, typically under 10% [24, 54]. For instance, Quyam et al. [24] detected LGE in only 2 of 30 long-term CCS (7%) more than 25 years post-treatment. The low prevalence observed in our cohort is consistent with these reports and may further reflect contemporary treatment regimens with reduced cumulative anthracycline exposure, careful CCS stratification, and systematic long-term cardiologic surveillance.

No significant prolongation of the global native T1, as well as no increase in ECV, was found in CCS in our study, in concordance with other studies [24, 27]. Likewise, some works support the diagnostic value of T1 mapping in detecting cardiotoxicity in CCS [18, 19, 27, 28], and prolonged native T1 as well as increased ECV have also been reported [18, 20–22, 25]. These differences between the studies may be mainly related to timing after therapy, as most studies assessed survivors within five years of treatment, while our cohort was evaluated approximately 12 years post-therapy. Another factor that could influence T1 or ECV findings is cumulative anthracycline exposure; however, given that our cohort’s doses were comparable to those reported in previous studies, it is unlikely that dose differences explain the observed discrepancies [20–24, 27]. While these factors likely contribute to the observed discrepancies, other unmeasured or study-specific variables may also play a role. Nonetheless, our study contributes to understanding the evolution of ECV in CCS cohorts, as our findings suggest that ECV levels in long-term CCS approximate those of control groups. However, this idea requires validation in prospective studies.

Unlike other LV functional and structural parameters, such as reduced myocardial mass or decreased LVEF, which show a clear association with cumulative anthracycline dose, no relationship was found between T1 longitudinal native values and ECV in our study. Even when compared with findings from other studies, this relationship is not visible. For instance, Harries et al. [20] observed significant changes in native T1 and ECV in a cohort exposed to similar cumulative anthracycline doses as our CCS (237 ± 83 mg/m^2^ versus 231.7 ± 92.0 mg/m^2^).

Overall, our findings support the concept that although overt cardiac dysfunction is uncommon in CCS during early adulthood, subtle alterations in systolic function and myocardial structure can be identified using sensitive imaging modalities, such as CMR. Moreover, circulating blood biomarkers may further enhance early detection and stratification when structural or functional myocardial abnormalities are present. In our anthracycline-treated survivors, biomarkers reflecting oxidative stress (MPO, IMA) and endothelial activation (bigET-1) were elevated, and CMR revealed differences compared to controls. Likewise, TM, IMA, and MPO correlated with some CMR measures, reflecting subclinical variations in myocardial structure and function rather than overt damage. Taken together, these findings are hypothesis-generating and require further research. Future longitudinal studies, ideally with larger cohorts, are needed to determine whether our findings can reliably predict cardiotoxicity and to establish their value in long-term cardiac risk stratification in CCS.

The study has several limitations. As a single-center project, the results may be influenced by local institutional practices and center-specific bias, potentially limiting generalizability. Two separate control groups were used for CMR and laboratory analysis. This approach was selected as a compromise to avoid the ethical concerns associated with administering contrast agents to healthy volunteers. Additionally, baseline values prior to oncological treatment were not available for either laboratory or imaging assessments, making it impossible to exclude the presence of pre-existing subclinical impairment entirely. In the case of oxidative stress markers, it is also challenging to determine whether their elevation represents a cause or a consequence of endothelial dysfunction.

## Conclusion

Our study demonstrates that while overt cardiac dysfunction is rare among CCS, subtle structural and functional myocardial changes may persist long after treatment. The combination of CMR and circulating biomarkers reflecting endothelial dysfunction and oxidative stress may offer complementary insights into subclinical cardiotoxicity and could help refine long-term cardiovascular risk assessment in this population.

## Supplementary Information

Below is the link to the electronic supplementary material.

Supplementary Material 1

## Data Availability

The data that support the findings of this study are not openly available due to reasons of sensitivity and are available from the corresponding author upon reasonable request.

## Abbreviations

ABSU: Absorbance unit
bigET-1: Big endothelin-1
BMI: Body mass index
BSA: Body surface area
CCS: Childhood cancer survivors
CCS-A: Childhood cancer survivors treated by anthracyclines
CHF: Chronic heart failure
CMR: Cardiac magnetic resonance
CG: Control group
CV: Coefficient of variation
ECV: Extracellular volume
EDTA: Ethylenediaminetetraacetic acid tripotassium salt dihydrate
EDV: End-diastolic volume
EF: Ejection fraction
ELISA: Enzyme-linked immunosorbent assay
ESV: End-systolic volume
I: Indexed
IMA: Ischemia-modified albumin
ISP: IntelliSpace portal
IR-TFE: Inversion-recovery turbo field echo sequence
LGE: Late gadolinium enhancement
LV: Left ventricle
MAPSE: Mitral annular plane systolic excursion
MM: Myocardial mass
MOLLI: Modified Look-Locker inversion recovery
MPO: Myeloperoxidase
NT-proBNP: N-terminal pro-B-type natriuretic peptide
PSIR: Phase-sensitive inversion recovery
RV: Right ventricle
SAX: Short-axis
SSFP: Steady-state free precession
SV: Stroke volume
T1: T1 longitudinal relaxation time
TM: Thrombomodulin
