# Supplementary material for: Long-term Subclinical Cardiotoxicity of Modern Cardiotoxic Treatment Protocols in Childhood Cancer Survivors Assessed by Cardiovascular Magnetic Resonance T1 Mapping and Circulatory Biomarkers
Source: Cardiovasc Toxicol. 2026 Jan 28;26(2):23. doi: 10.1007/s12012-026-10098-8 (PMC12852242; doi:10.1007/s12012-026-10098-8)
Supplement: Supplementary file 1 — Supplementary Material 1 [file 12012_2026_10098_MOESM1_ESM.docx]

**Supplementary Table 1 Left ventricular functional and volumetric parameters for CCS according to chest radiotherapy treatment**

| **Parameter** | **CCS treated with chest radiotherapy  (n =47)** | **CCS not treated with chest radiotherapy  (n = 70)** | **p-value** |
| --- | --- | --- | --- |
| LVEF (%) | 58.3 (4.4) | 59.4 (6.1) | 0.270 |
| LVEDV (ml) | 118.4 (26.5) | 141.0 (30.0) | **<0.001** |
| LVESV (ml) | 49.6 (13.6) | 57.5 (16.5) | **0.005** |
| LVSV (ml) | 68.8 (14.9) | 83.6 (18.1) | **<0.001** |
| LVMM (g) | 62.0 (21.9) | 79.2 (24.9) | **<0.001** |
| LVEDVi (ml/m^2^) | 67.5 (12.0) | 77.8 (13.3) | **<0.001** |
| LVESVi (ml/m^2^) | 28.2 (6.6) | 31.7 (8.2) | **0.012** |
| LVSVi (ml/m^2^) | 39.2 (6.9) | 46.1 (8.8) | **<0.001** |
| LVMMi (g/m^2^) | 34.9 (9.2) | 42.6 (11.9) | **<0.001** |
| MAPSE (mm) | 12.1 (1.5) | 12.8 (1.7) | **0.018** |

Variables are expressed as mean (standard deviation), p-values correspond to Welch’s t-test. Abbreviations: CCS, childhood cancer survivors; n, total number of subjects; EF, ejection fraction; EDV, end-diastole volume; ESV, end-systole volume; i, indexed; LV, left ventricle; MM, myocardial mass; MAPSE, mitral annular plane systolic excursion; SV, stroke volume.
